# Nearly perfect near-infrared luminescence efficiency of Si nanocrystals: A comprehensive quantum yield study employing the Purcell effect

**DOI:** 10.1038/s41598-019-47825-x

**Published:** 2019-08-02

**Authors:** J. Valenta, M. Greben, S. A. Dyakov, N. A. Gippius, D. Hiller, S. Gutsch, M. Zacharias

**Affiliations:** 1 Charles University, Faculty of Mathematics & Physics, Department of Chemical Physics & Optics, Prague, Czechia; 2 Skolkovo Institute of Science and Technology, Moscow, Russia; 3 Laboratory of Nanotechnology, IMTEK, Faculty of Engineering, Albert-Ludwigs Universität Freiburg, Freiburg im Breisgau, Germany; 4 Research School of Engineering, Australian National University, Canberra, Australia

**Keywords:** Quantum dots, Nanophotonics and plasmonics, Surfaces, interfaces and thin films

## Abstract

Thin layers of silicon nanocrystals (SiNC) in oxide matrix with optimized parameters are fabricated by the plasma-enhanced chemical vapor deposition. These materials with SiNC sizes of about 4.5 nm and the SiO_2_ barrier thickness of 3 nm reveal external quantum yield (QY) close to 50% which is near to the best chemically synthetized colloidal SiNC. Internal QY is determined using the Purcell effect, i.e. modifying radiative decay rate by the proximity of a high index medium in a special wedge-shape sample. For the first time we performed these experiments at variable temperatures. The complete optical characterization and knowledge of both internal and external QY allow to estimate the spectral distribution of the dark and bright NC populations within the SiNC ensemble. We show that SiNCs emitting at around 1.2–1.3 eV are mostly bright with internal QY reaching 80% at room temperature and being reduced by thermally activated non-radiative processes (below 100 K internal QY approaches 100%). The mechanisms of non-radiative decay are discussed based on their temperature dependence.

## Introduction

Silicon nanocrystals (SiNC) embedded in silicon dioxide (SiO_2_) have been shown to provide high photoluminescence (PL) quantum yield (QY) of the order of 20% which is size tunable in the spectral region from orange to near infrared (NIR), i.e. about 650–1100 nm^1^. Such quality can be potentially exploited to provide photon conversion in lighting and photovoltaic devices^2^.

The Purcell effect is named after E. M. Purcell who with coworkers demonstrated that the probability of spontaneous radiative transition can be modified by the surrounding medium^3^. The probability of spontaneous decay of an emitter is defined by the local density of optical states (LDOS) that can be tuned by putting an emitter in a cavity. The ratio of the cavity-modified and the non-modified radiative decay rates is known as *Purcell factor* (PF). The emission rate of NCs can be influenced by the LDOS engineering based on the environmental dependence of the intrinsic radiative rate. Mesoscopic systems like microcavities, photonic crystals, thin layers and interfaces could be employed. The coupling strength between the emitter and the cavity is divided into the weak or strong coupling regime. The weak coupling regime, when photons are emitted from the considered system faster than the interaction time between the emitter and the cavity, is most likely to be observed in SiNCs^4^.

During the last decades the plasma-enhanced chemical vapor deposition (PE-CVD) proved to provide highly controllable and reproducible multilayer (ML) structures of alternating layers of SiNCs and silicon-oxynitride (SiO_x_N_y_) or pure silica (SiO_2_)^5^. In this paper we provide a comprehensive study of both external and internal luminescence quantum yield (IQY, EQY) of SiNC/SiO_2_ multilayers. The study shows that a maximum EQY close to 50% is achieved in well passivated multilayers with SiNC sizes of about 4.5 nm and the SiO_2_ barrier thickness of 3 nm (or larger). IQY is determined using the Purcell effect, i.e. modifying radiative decay rate by the proximity of a high index medium in a special wedge-shape sample. For the first time we performed these experiments at variable temperatures. The complete optical characterization and knowledge of both IQY and EQY allow to estimate the spectral distribution of the dark and bright NC populations within the SiNC ensemble. We show that SiNCs emitting at around 1.2–1.3 eV are mostly bright with IQY reaching 80% at room temperature and being reduced by thermally activated non-radiative processes (below 100 K IQY approaches 100%). The mechanisms of non-radiative decay are discussed based on their temperature dependence.

## Results

### Theoretical background – the Purcell effect and the decay rates

The Purcell factor is defined as a ratio of cavity modified and non-modified radiative decay rates

1 $${F}_{p}=\frac{{\Gamma }_{r}^{cav}}{{\Gamma }_{r}}$$

Semiclassical theory of radiation states that the quantum mechanical radiative decay rate is proportional to the total power dissipated by classical dipole emitters (see Supplementary Materials for details)

2 $${\Gamma }_{r}=\frac{P}{\hslash \omega }$$

This fact brings us to the alternative formula for Purcell factor

3 $${F}_{p}({\rm{\omega }})=\frac{{P}^{cav}(\omega )}{P},$$

where *P*^*cav*^(ω) is the total power dissipated by dipole emitters inside the structure and *P* is a total power dissipated by the same dipoles in their infinite host matrix:

4 $${P}^{cav}(\omega )=\underset{0}{\overset{\infty }{\iint }}p(\omega ,{k}_{x},{k}_{y})d{k}_{x}d{k}_{y}$$

5 $$P=\frac{{j}_{0}^{2}{\omega }^{2}}{3{c}^{3}}\sqrt{\varepsilon }$$

In Eqs 4 and 5 *ε* is the dielectric permittivity of host matrix of SiNCs, ω is the angular frequency of electromagnetic oscillations, *k*_*x*_ and *k*_*y*_ are the x and y components of photon wavevector, *j*_0_ is the amplitude of electric current oscillation, *c* is the speed of light. The spectral power density *p*(*ω*, *k*_*x*,_ *k*_*y*_) depends on the dipoles orientation and its position in the structure and can be found by the method of source terms for dipole emission^6,7^.

In our calculations, we place the point dipole emitters in the center of emitting layer with SiNCs and assume a chaotic dipoles orientation. The detailed description of the multilayer structure is given below. Depending of the spacer thickness, the excited SiNCs may decay with the emission of light, which (i) is coming out into the far field; (ii) is coupled to guided or lossy waves in SiNCs layer; (iii) is absorbed by the substrate. The entire contribution of these decay mechanisms determines the radiative decay rate, which is completely described by Eq. (4). There is also a non-radiative decay rate, which is not modified by the LDOS and which occurs without a photon emission. The total decay rate in a cavity is a sum of radiative and non-radiative decay parts:

6 $${{\rm{\Gamma }}}_{tot}(x)={F}_{p}({\rm{x}}){\Gamma }_{r}+{\Gamma }_{nr},$$

where *Γ*_*nr*_ is the constant non-radiative decay rate and *F*_*p*_(*x*) is the PF which is a function of dipole position along the sample length. The IQY is defined as

7 $$\eta =\frac{{{\rm{\Gamma }}}_{r}}{{{\rm{\Gamma }}}_{r}+{{\rm{\Gamma }}}_{nr}}$$

that, by definition, describes dipoles placed in a homogeneous dielectric *ε* without boundaries (and then PF is apparently equal to 1).

By fitting Eq. (6) to experimental data, we obtain *Γ*_*r*_ and *Γ*_*nr*_ independently which enables us to determine the internal quantum efficiency.

### Fabrication and characterization of SiNC multilayer samples

The samples were deposited as alternating layers of 4.5-nm-thick silicon-rich silicon oxide (SRO; SiO_x_, x = 0.9) and stoichiometric SiO_2_ (3 nm) on fused silica or silicon substrates by PECVD. The samples were consequently annealed in high purity N_2_ in order to achieve phase separation between Si and SiO_2_ and then additionally passivated by annealing in H_2_. (See Methods for more details).

**Figure 1:**
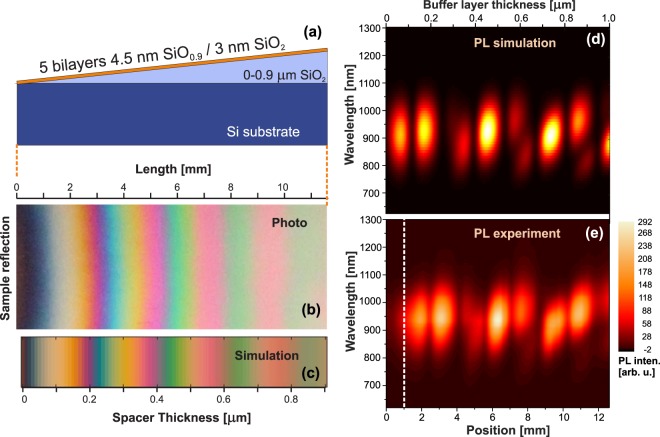
(**a**) The schematic cross-section of the wedge sample on Si substrate, (**b**) the reflectance photograph and (**c**) simulated image, (**d**) simulated and (**e**) measured PL maps of the wedge sample - the position at 1 mm corresponds roughly to zero thickness of the wedge spacer.

For the EQY measurements, 50 bilayers were deposited on a fused silica substrate (in order to reach good absorption for precise calculation of EQY), while the sample for IQY measurements contains only 5 bilayers deposited on a Si substrate with a wet-thermal SiO_2_ spacer layer of the form of a wedge with thickness ranging from 900 to 0 nm (made by slant etching in buffered hydrofluoric acid), see Fig. 1. Such low number of bilayers is required in order to have an approximately uniform PF throughout the effective optical layers.

The wedge sample was characterized by spectroscopic ellipsometry at different positions on the sample in order to obtain the optical parameters for theoretical simulations. Experimental and simulated reflectance and photoluminescence changes along the wedge are compared in Fig. 1. The wavy PL intensity (Fig. 1d,e) is due to interference of both the excitation laser (405 nm) and the emitted luminescence being reflected on the air/silica and silica/Si interfaces. The simulations are in excellent agreement with the experimental data. (Note: The edge part, about 1 mm, of the wedge sample has intentionally no buffer layer (it is fully etched out). This is necessary since the PECVD deposition of the SiNC-layers is inherently affected by edges, which results in inhomogeneity – therefore, the measurement starting point must be shifted from the edge, see the dashed line in Fig. 1e).

**Figure 2:**
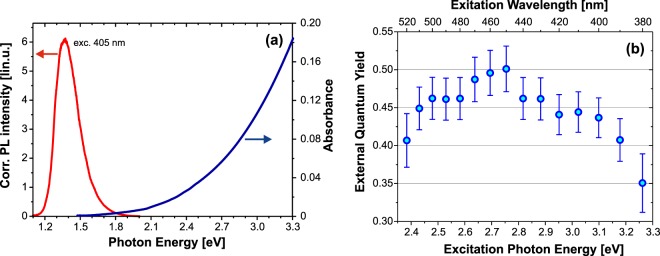
(**a**) Broad range PL and absorbance spectra of the 50 bilayer sample and (**b**) EQY excitation spectrum (both at room temperature).

Absorbance and PL spectra (405 nm excitation) of the EQY sample measured over a broad spectral range (combining the visible and NIR cameras) are shown in Fig. 2a.

EQY was measured over the available excitation range (limited by the system transmittance in the UV and by the low absorbance of the sample in the green spectral region) using the methodology described in^8^ where the reference measurements were done with the empty integrating sphere. The peak EQY around 50% is obtained for blue excitation around 450 nm (Fig. 2b) which is the highest reported value for SiNCs embedded in oxide matrix. (Note, for chemically synthesized colloidal Si NCs EQY of about 60% or even more was recently reported by many groups^9^, however, with peak values which are usually shifted to shorter emission wavelengths).

**Figure 3:**
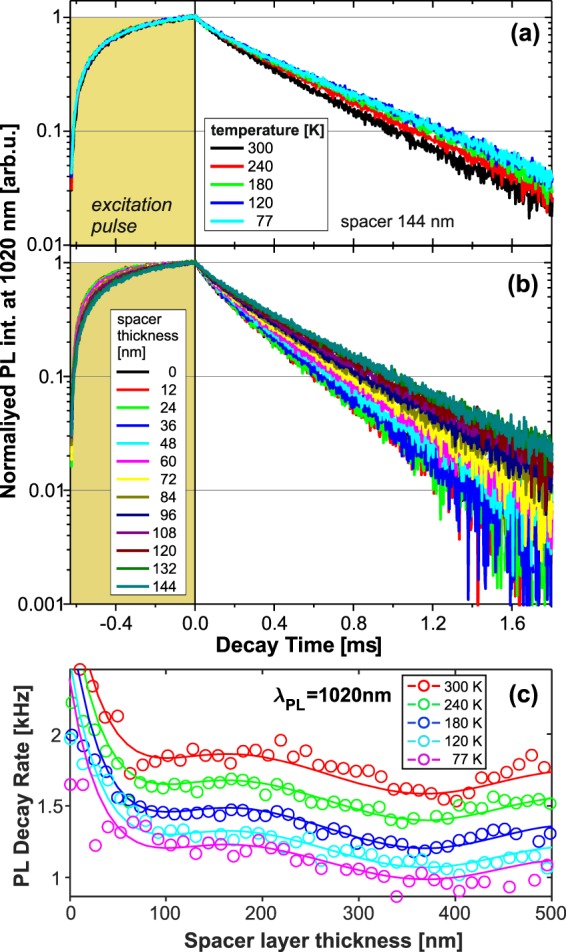
Examples of measured onset and decay kinetics under modulated excitation: (**a**) Temperature changes of PL decay at 1020 nm at position with 144 nm spacer; (**b**) PL decay changes for the first 12 positions (spacer 0 to 144 nm) detected at 1020 nm and room temperature (300 K). (**c**) The average decay rate at 1020 nm as function of the spacer thickness and temperature; the solid lines are the fits using Eq. (6). The five fitted curves reveal negligible changes of the wavy shape being just shifted to higher rates with increasing temperature. This indicates that the temperature is affecting almost exclusively the non-radiative decay rates.

PL decay kinetics were excited by approximately rectangular pulses several tens of microseconds long (see Fig. 3a,b) in order to obtain high signal amplitudes (almost reaching the steady-state level) and to optimize the signal/noise ratio in a reasonable experimental time^10^. The excitation power density must be kept well below the typical saturation level of 1 W/cm^2^ (we commonly used ~0.1 W/cm^2^). The average decay times were obtained by fitting with combination of two stretched-exponential or three mono-exponential functions and calculated as the intensity average^11^.

### Spectral and temperature distribution of radiative and non-radiative rates and IQY

PL decay kinetics were measured at 82 or 56 points (at RT and low-T, respectively) on the sample along the changing wedge thickness using a manual micrometer-screw stage which shifted the whole cryostat. This was done for eight emission wavelengths (from 740 to 1060 nm) and five temperatures (300, 240, 180, 120 and 77 K), which represents together about 2500 decay traces measured at tentatively selected conditions to obtain an as-good-as-possible S/N ratio in order to reduce uncertainty of the deduced mean decay rates and the consequent simulations.

**Figure 4:**
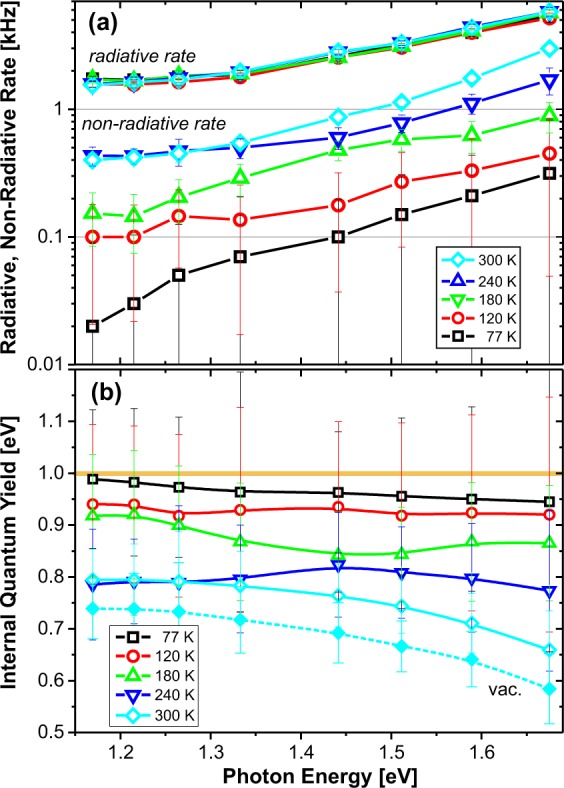
(**a**) Radiative (topmost overlapped data) and non-radiative rates as function of emission wavelength and temperature. (**b**) IQY calculated from the rates presented in the panel (a), the radiative rates in the material are considered, except the bottom data (filled cyan diamond-symbols with dashed line) which shows IQY at RT in vacuum.

The variation of average decay rates along the wedge for different wavelengths and temperatures was fitted using Eq. (6) which gives us radiative and non-radiative rates and consequently IQY (Fig. 4). While the radiative rates reveal very small temperature changes (within the studied range), the non-radiative rates strongly increase with temperature (Fig. 4a). Consequently, the IQY obtained as the ratio of radiative and total rates is also temperature dependent (Fig. 4b) and slightly decreasing towards shorter wavelengths. The highest IQY is found around 1000 nm (1.2 eV): 74% and almost 100% at room temperature and 77 K, respectively.

Then, we analysed the temperature dependence of non-radiative rates using Arrhenius plots and fitting by the following equation

8 $${{\rm{\Gamma }}}_{nr}={{\rm{\Gamma }}}_{0}+k\cdot {e}^{-\frac{{E}_{A}}{{k}_{B}T}}$$

**Figure 5:**
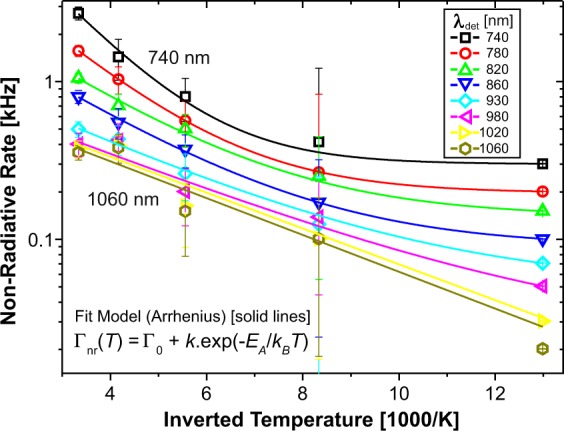
The Arrhenius plot of the natural logarithm of non-radiative rates vs. inverse temperature. The increasing deviation from linear shape with decreasing emission wavelength indicates the presence of a thermally independent rate (constant term in Eq. (8)).

**Figure 6:**
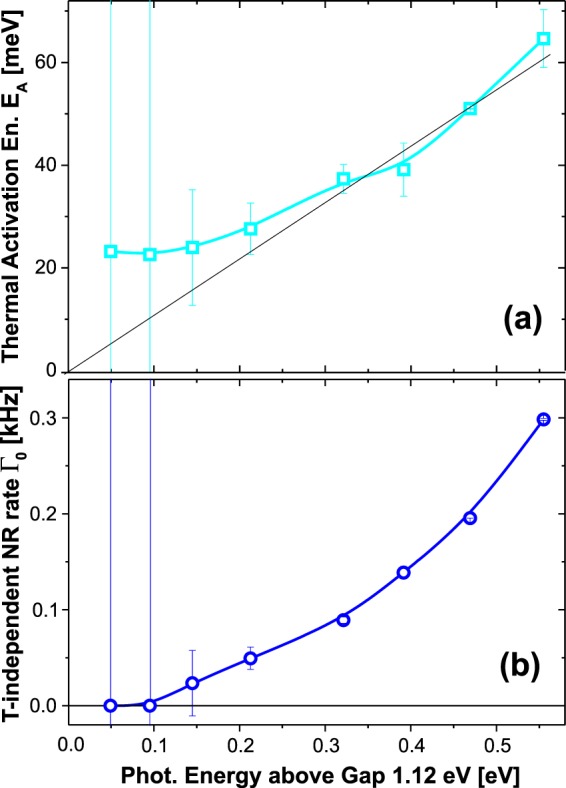
Emission photon energy dependence of the thermal activation energy (**a**) and the T-independent non-radiative rate (**b**).

Here, in addition to the thermally activated process with activation energy *E*_*A*_, we had to introduce a T-independent rate *Γ*_0_ (Fig. 5). The spectral distributions of the fitting parameters *E*_*A*_, and *Γ*_0_ are presented in Fig. 6. The activation energy increases with photon energy from about 23 to 62 meV, while the constant NR rate appears only above 1.2 eV and increases up to 0.3 kHz. Therefore, the T-activated and T-independent processes dominate at the long and short wavelength edge of the PL spectrum, respectively.

### Determining fractions of bright and dark NCs at room temperature

The IQY result described above may be compared with EQY of an equivalent sample deposited on silica substrate and containing 50 bilayers. According to results presented in Fig. 2b we take EQY 43% for excitation at 405 nm.

The fraction of bright NCs *F*_*B*_ in an ensemble is commonly retrieved via the simple relation^12^

9 $${F}_{B}=\frac{{\eta }_{E}}{{\eta }_{I}},$$

where the integral or PL peak value is taken. In our case we found an average IQY about 77%, which gives *F*_*B*_ about 0.56, i.e. more than half of NCs in the ensemble are bright.

However, we know that the size distribution *D* and the spectral (resp. size) changes of parameters describing the PL spectrum are very important. Therefore, we can expect that the distribution of bright NCs will strongly change with spectral position.

The PL spectral shape is described by the following equation

10 $${I}_{PL}(\lambda )=K\cdot {{\rm{\Phi }}}_{ex}\cdot \sigma (\lambda )\cdot {D}_{E}(\lambda )\cdot {\eta }_{I}(\lambda )\cdot {d}_{B}(\lambda ),$$

where the first four terms describe the excitation rate (Φ_ex_ is excitation photon flux, *σ* is absorption cross-section (ACS) and *D*_*E*_ is the NC size distribution transformed to emission photon energy scale) and the last two terms describe the probability that excited NCs will emit photons (*η*_*I*_ is the internal QY and *d*_*B*_ the spectral distribution of the relative population of bright NCs). The constant factor *K* represents mostly the absolute value of coupling of emitted photons into the detection path and efficiency of detection (the set-up is calibrated for the relative spectral sensitivity only). This constant can be put together with other scaling factors, i.e. the excitation photon flux Φ_ex_ and possibly also the uncertainty of absolute value of the size distribution *D*_*E*_ (in fact all distributions can be just relative, normalized). We can also transform the wavelength to photon energy and express the searched *d*_*B*_ as

11 $$const\cdot {d}_{B}(E)={I}_{PL}(E)/(\sigma (E)\cdot {D}_{E}(E)\cdot {\eta }_{I}(E)),$$

which should hold for any small interval *dE* within the PL spectrum and allows us to calculate the distribution of *d*_*B*_ (with unknown scale) as all necessary spectral distributions of quantities are known: The IQY is determined in this paper and ACS is taken from our previous work^13^. The size distribution *D*_*E*_ is taken from^14^ with the size scale transformed into emitted photon energy using the theoretical relation for the excitonic gap of SiNCs derived by Luo *et al*.^15^ and shifted by −0.2 eV, as the matrix embedded SiNCs reveal such shift^16^ (probably due to strain).

Then, the proper scaling of *d*_*B*_ is obtained using the external QY, which is defined as the ratio of emission rate to absorption rate of the whole NCs ensemble, i.e.

12 $${\eta }_{E}=\frac{{{\rm{\Gamma }}}_{em}}{{{\rm{\Gamma }}}_{ex}}={\int }_{em.sp.}\frac{\sigma (\lambda ){D}_{E}(\lambda ){\eta }_{I}(\lambda ){d}_{B}(\lambda )}{\sigma (\lambda ){D}_{E}(\lambda )}d\lambda ={\int }_{em.sp.}{\eta }_{I}(\lambda ){d}_{B}(\lambda )d\lambda .$$

**Figure 7:**
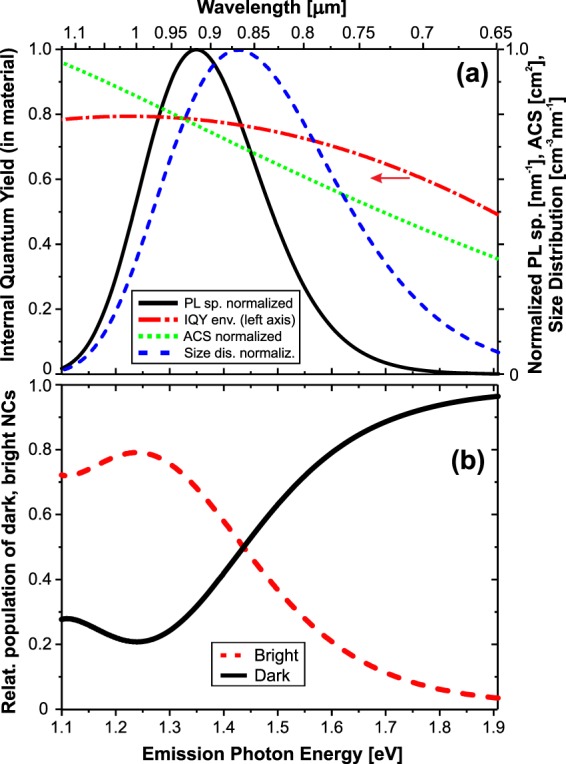
(**a**) Components used to calculate the spectral distribution of the fraction of bright nanocrystals using Eq. (11). (**b**) The relative distribution of bright and dark NCs in the SiNCs ensemble.

The result is shown in Fig. 7b yielding a majority of bright population above 860 nm with the peak of 0.8 around 1000 nm (1.24 eV) with reduction toward both lower and higher photon energies. Especially the strong decrease at higher photon energies is significant. The relative population of dark NCs is obtained simply as 1 − *d*_*B*_.

## Discussion

The main results of this paper, presented in Figs 4 to 7, reveal high efficiency of luminescence from SiNCs in oxide matrix in the near-IR spectral region. At room temperature, almost all NCs emitting around 1.3 eV are bright and their IQY is above 75% - limited just by the thermally activated non-radiative decay (activation energy around 23 meV, increasing to ~65 meV in smaller SiNCs). This loss channel is most probably due to carrier escape from a Si core to nearby traps at the Si/SiO_2_ interface. Similar values of thermal activation energy of ~60–100 meV and ~36 meV were found in studies of temperature changes of PL decay rates in both the ML ensembles of SiNCs^17^ as well as single SiNCs with oxide passivation^18^, respectively.

The decrease of the bright NC population closer to the bulk Si band gap may be related to increasing probability of having a dangling bond defect in these large nanocrystals. In our recent paper we estimated the surface density of dangling bonds (using changes of EQY upon H-annealing, passivation) to be 2.5 × 10^12^ cm^−2^^19^. Therefore, large NCs with a radius of 10 nm should have about 5 dangling bonds. Even if the H-passivation works very well, it may happen that part of such big NCs will have some remaining dangling bonds, which efficiently quench the PL. It may be possible to invent some more efficient passivation procedure in future.

The more difficult situation is towards small SiNCs where both the IQY and the bright population decreases. Consequently, in the green spectral region, the PL is vanishing. Indeed, this effect was observed already in porous Si, where the PL peak can be tuned (e.g. by prolonged etching or oxidation) from red to yellow with continuous shortening of decay time. However, the PL in the green region vanishes and eventually a different PL band appears in the blue-UV region with many times faster decay^20^ (this F-band is sometimes interpreted to be due to defects but also due to emerging direct transitions^21^). Similarly, the EQY decreases towards the green region was reported for chemically synthesized organically passivated SiNCs^22^. The present work shows that IQY reduces from about 80 to 65% when going from NIR/red to yellow spectral region, which is due to an increasing non-radiative rate, especially its temperature independent part. This suggests the mechanism of resonant tunnelling to oxide defect states that come to a resonance by widening of the NC band gap and with spreading of quasiparticle wavefunctions out of small NCs (a similar explanation was used by Yu *et al*. and called the finite-confinement model^23^). The increase of the dark nanocrystal fraction is even stronger than the reduction of IQY. As the probability to have dangling bonds on small NCs is low, there must be some other types of defects appearing with increasing curvature of the Si/silica interface – e.g. the distorted Si-Si and Si-O-Si bonds^24^. If this was the reason for inefficient green PL, then one can hardly think about any improvement other than some kind of strain engineering.

The difference of EQY and IQY was interpreted in this paper to be due to the presence of absorbing but non-emitting NCs – dark NCs. However, there is an important question about the real nature of such “dark nano-matter”. Is a fraction of NCs permanently dark/bright or do some of the NCs switch between bright and dark state, i.e. show some blinking? It is well known and described in literature that individual Si NCs with oxide passivation can blink^25^, but we have no such information for our ML samples. If such blinking SiNCs exist (let us call them grey or semi-bright), we cannot reveal them from the present experiments. Recently, Pevere *et al*. presented experiments on single SiNCs and proposed a model of blinking due to carrier tunnelling to the resonant traps which leads to delayed PL and reduction of IQY^26^. Similar effects may occur also in our ML samples, however, our comparison of IQY and EQY only give the long-time average of that fraction of the SiNCs ensemble which is emitting or non-emitting at any moment.

Regardless to the above mentioned losses, the SiNC/SiO_2_ multilayers with NC size around 4.5 nm and PL peak around 950 nm have excellent performance (EQY~50% and IQY~80%) compared to any fluorophore emitting in this region, e.g. IR 140 dye or Styril 14 (EQY~14% and 9%, respectively^27^). Some other nanocrystals can have comparable EQY, e.g. suspensions of PbS NCs with diameters of about 3 nm emitting in this region and having EQY in the range 20–50%^28^ (their EQY is, however, significantly reduced when placed in solid state matrix^29^ and the material itself is toxic). Therefore, thin SiNCs multilayers may find application as stable and efficient NIR luminescing layers with large Stokes shift – for example, we have demonstrated their application for advanced calibration of a two-detector micro-spectroscopy set-up^9^.

Finally, our optimized method to study internal quantum yield of thin-layer materials at variable temperatures employing the Purcell effect is not restricted to the SiNC ML but is broadly applicable to various light-emitting nanostructured materials.

## Methods

### Sample fabrication

Alternating layers of 4.5-nm-thick silicon-rich silicon oxide (SRO; SiO_x_, x = 0.9) and stoichiometric SiO_2_ (3 nm) were deposited by the plasma-enhanced chemical vapor deposition (PE-CVD) on fused silica or silicon substrates. Below and above the multilayer stack, 3 nm and 50 nm of SiO_2_ were deposited as a buffer and capping layer, respectively. The samples were consequently annealed in a quartz tube furnace at 1100 °C for 1 h in high purity N_2_ in order to achieve phase separation between Si and SiO_2_ (i.e. forming Si nanocrystals). The samples were additionally passivated by annealing in H_2_ at 500 °C for 1 hour. Further details of the sample preparation as well as structural properties of the NC samples are given in our recent paper^30^.

### Optical spectroscopy methods

Luminescence spectra were measured using our home-build micro-spectroscopy set-up based on an inverted optical microscope (Olympus IX-71). Wide-field excitation is performed in the epifluorescence configuration by a 405-nm diode laser. The emitted signal is divided by a short-pass dichroic beam-splitter and imaged on the input slits of two detection spectrographs. The visible spectral range (~350–1100 nm) is detected by a 30-cm imaging spectrograph with a back-thinned LN-cooled CCD camera (Spec-10:400B, Princeton Instruments), while the NIR wavelengths (~950–1640 nm) are detected by a 50-cm imaging spectrograph with an InGaAs camera (NIRvana, Princeton Instruments). The sample is placed in a micro-cryostat (Janis ST-300) on an x-y manual stage and an objective lens with correction to the cryostat window is used (Nikon PlanFluor ELWD 40×/0.6). Spectral sensitivity of the complete apparatus is calibrated over the broad VIS-NIR spectral range using a 45-W tungsten halogen lamp (the secondary radiation standard, Newport Oriel)^31^.

PL decay kinetics are measured in the same micro-spectroscopy set-up using the side exit ports of the spectrometers where the photon-counting photomultipliers (Hamamatsu H11526–20-NF and H10330A-45, respectively) detect the selected spectral region and the signal is send to a pair of multichannel scaler cards (Becker & Hickl MSA-300). The excitation pulse is obtained from the continuous 405 nm laser beam using a silica acousto-optical modulator. We devoted special attention to the effects of excitation pulse length and intensity on the correct assessment of the decay times, see our recent paper and review^10,11^.

EQY was measured using an integrating sphere (d = 10 cm, Spectraflect coating) with the tunable excitation provided by a laser driven light source (LDLS, EQ-99X from Energetiq) coupled to a 15-cm monochromator. The excitation and emission signals were guided to and from the integrating sphere by silica fiber bundles. The emitted signal from the waveguides is imaged to the micro-spectroscopy set-up (described above) using a home-build coupler.

## Supplementary Information

Supplementary Information
