# Supplementary material for: Nearly perfect near-infrared luminescence efficiency of Si nanocrystals: A comprehensive quantum yield study employing the Purcell effect
Source: Sci Rep. 2019 Aug 2;9:11214. doi: 10.1038/s41598-019-47825-x (PMC6677743; doi:10.1038/s41598-019-47825-x)
Supplement: Supplementary file 1 — Supplementary Information [file 41598_2019_47825_MOESM1_ESM.pdf]

Supplementary materials to the article

Nearly perfect near-infrared luminescence efficiency of Si nanocrystals: A comprehensive quantum yield study employing the Purcell effect

J. Valenta[1], M. Greben[1], S. A. Dyakov[2], N. A. Gippius[2], D. Hiller[3,4], S. Gutsch[3], M. Zacharias[3]*

[1] *Charles University, Faculty of Mathematics & Physics, Department of Chemical Physics & Optics, Prague, Czechia*

[2] *Skolkovo Institute of Science and Technology, Russia*

[3] *Laboratory of Nanotechnology, IMTEK, Faculty of Engineering, Albert-Ludwigs Universität Freiburg, Germany*

[4] *Research School of Engineering, Australian National University, Canberra, Australia*

To find the relation between the radiative decay and the emitters dissipated power, we consider the classical model of dissipating dipole with energy *E* and total power flux *P*. The total energy stored in the dipole is proportional to the square of amplitude of the dipole moment p:

$$E = \alpha p^2, \tag{1}$$

where α is the proportionality factor. Here we assume that the dipole is not infinitely small. This important assumption solves the problem of divergent energy density in the proximity of the dipole. The dipole's dissipated power is also proportional to the square of amplitude of the dipole moment:

$$P = \beta p^2, \tag{2}$$

where β is the proportionality factor. Since $\dot{E} = -P$, we obtain the following differential equation

$$2\alpha\dot{p} = -\beta p \tag{3}$$

with solution

$$p(t) = p_0 e^{-\frac{\beta}{2\alpha}t}. \tag{4}$$

Hence, the energy and the power flux decay as

$$E(t) = \alpha p_0^2 e^{-\frac{\beta}{\alpha}t}, \qquad P(t) = \beta p_0^2 e^{-\frac{\beta}{\alpha}t} \tag{5}$$

with the same decay constant Γ

$$\Gamma = \frac{\beta}{\alpha} = \frac{P}{E}. \tag{6}$$

Replacing *E* with the energy of emitted photon $\hbar\omega$ we obtain

$$\Gamma = \frac{P}{\hbar\omega}. \tag{7}$$
